# CMOS Image Sensor with a Built-in Lane Detector

**DOI:** 10.3390/s90301722

**Published:** 2009-03-12

**Authors:** Pei-Yung Hsiao, Hsien-Chein Cheng, Shih-Shinh Huang, Li-Chen Fu

**Affiliations:** 1 Department of Electrical Engineering, National University of Kaohsiung, Kaohsiung, Taiwan, ROC; 2 Department of Electronic Engineering, Chang Gung University, Tao-Yuan, Taiwan, ROC; E-Mail: Jason.cheng@genesyslogic.com.tw; 3 Department of Computer and Communication Engineering, National Kaohsiung First University of Science and Technology, Kaohsiung, Taiwan, ROC; E-Mail: powwhuang@gmail.com; 4 Department of Computer Science and Information Engineering, National Taiwan University, Taipei, Taiwan, ROC; E-Mail: lichen@ntu.edu.tw

**Keywords:** Image sensor, CMOS, photodiode, current-mode, lane detection, peak-finding algorithm, Gaussian filter, intelligent transportation systems

## Abstract

This work develops a new current-mode mixed signal Complementary Metal-Oxide-Semiconductor (CMOS) imager, which can capture images and simultaneously produce vehicle lane maps. The adopted lane detection algorithm, which was modified to be compatible with hardware requirements, can achieve a high recognition rate of up to approximately 96% under various weather conditions. Instead of a Personal Computer (PC) based system or embedded platform system equipped with expensive high performance chip of Reduced Instruction Set Computer (RISC) or Digital Signal Processor (DSP), the proposed imager, without extra Analog to Digital Converter (ADC) circuits to transform signals, is a compact, lower cost key-component chip. It is also an innovative component device that can be integrated into intelligent automotive lane departure systems. The chip size is 2,191.4 × 2,389.8 μm, and the package uses 40 pin Dual-In-Package (DIP). The pixel cell size is 18.45 × 21.8 μm and the core size of photodiode is 12.45 × 9.6 μm; the resulting fill factor is 29.7%.

## 1. Introduction

Over the last decade, Intelligent Transportation Systems (ITS) have received considerable attention. ITS covers lane detection, obstacle recognition, car following and other areas. Instead of a Personal Computer (PC) based system or an embedded platform system equipped with expensive high performance chip of Reduced Instruction Set Computer (RISC) or Digital Signal Processor (DSP), this study develops a low-cost, compact, and portable Complementary Metal-Oxide-Semiconductor (CMOS) imager to capture images and detect lanes in real-time in an intelligent automotive lane departure system. In another respect, the use of the single chip specific CMOS imager for visual lane detection makes it more difficult for competitors to copy and reverse engineer than the use of an embedded system platform loosely combined with a normal CMOS imager module. The single chip solution certainly insures a longer product life as well as a higher value-added profit in high-tech market. As far as we know, this idea and the proposed prototype chip have never been described in the literature.

Various vision-based lane detection algorithms [1–6] have been proposed in recent years. For example, Kluge and Lakshmanan [3] proposed the LOIS (Likelihood of Image Shape) lane detection algorithm, called the Metropolis algorithm. It detects lanes based on a stochastic optimization procedure even when shadows or broken lanes exist. However, Metropolis algorithm sometimes fails to reach a global maximum when the algorithm starts from a poor initial solution. Takahashi *et al.* [4] divided the parameter space of the lane model to generate the lane marking patterns and then applied the voting scheme to find the lane boundary. This algorithm does not guarantee a global optimum or satisfactory accuracy without huge computational resources. Moreover, both of these works are difficult to implement into hardware circuits. Broggi [5] presented an edge-based road detection algorithm, which is effective only for well-painted roads. In addition, adjusting the threshold to accommodate various weather conditions and traffic environments is difficult in Broggi’s approach.

Most CMOS imager designs have been proposed with focuses on high resolution, high dynamic range, and noise reduction, rather than for embedded integration for particular applications. Coulomb and Mohamad [7] proposed a current mode active pixel sensor for variable resolutions with offset and gain compensation to reduce Fixed Pattern Noise (FPN). Their FPN suppression scheme improves the ability to inhibit noise under different light conditions, such as darkness or brightness. However, they didn’t further explain how to develop a valuable System-on-Chip (SoC).

In the recent literature, only a few investigations have integrated edge detection and motion detection in a CMOS image sensor [8–10]. Typically, edge detection operation only computes the difference of intensity between adjacent pixels. These approaches tend to yield abrupt broken edges or non-single pixel edges that lead to poor quality of resultant edges caused by noise and inaccuracy when identifying the edge points. Yang *et al*. [10] proposed a 256 × 256 smart CMOS image sensor for line-based vision applications. They used the edge-based architecture to extract the feature points and then applied a histogram equalization based signal regularization approach to reduce the computational errors associated with an analogous circuit operation. However, this CMOS imager is more suitable for embedded motion detection than for automotive lane detection in smart vehicles.

**Figure 1.**
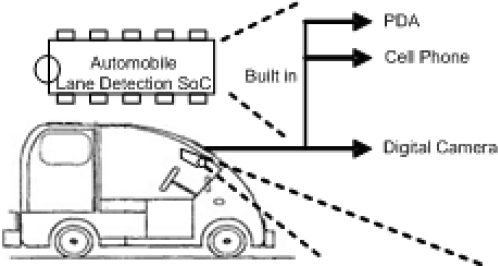
The automobile lane detector integrated with CMOS imager for use in ITS.

In this work, the developed CMOS imager, without using extra Analog to Digital Converter (ADC), circuits for signal transformation, is a single, low-cost, and compact chip for image capture and lane detection at the same time. The current industrial solution employs an embedded platform with Advanced RISC Machine based (ARM-based) CPU or DSP processor to support the computation intensity of lane detection algorithm. In this platform, large memory capacity [Random Access Memory (RAM) and flash Read Only Memory (ROM)], are required to run the real time Operating System (OS) and store the lane detection algorithm program. In our SoC-based approach, the lane detection algorithm is designed within the CMOS imager. Without the costs of ARM-based CPU (or DSP processor) and larger memories, the proposed imager should be a better low-cost and compact solution. Further, this innovative component device can be easily integrated in a SoC-based intelligent automotive lane departure warning system. The designed mixed signal CMOS imager is capable of capturing images as well as producing vehicle lane maps at the same time. Moreover, it can be easily integrated with currently existing consumer electronic devices, such as Personal Digital Assistance (PDA), cell phone, digital camera, etc. for real-time lane detection applications. Figure 1 shows how our proposed automobile lane detector with integrated CMOS imager could be used in ITS in the very near future.

This paper is organized as follows. Section 2 describes the embedded modified algorithm for vehicle lane detection. The proposed CMOS imager architecture and the developed analog circuit design for lane detection are introduced in Section 3. The circuit design includes two new types of pixel cells, dual Gaussian filters, the analog module for peak finding with auto-regulated threshold, and the lane point allocation in digital form. Section 4 presents the experimental results, and the final section reports our conclusions.

## 2. Vehicle Lane Detection Algorithm

Lane detection is one of the important and fundamental issues in an intelligent transportation system. In this paper, the lane detecting algorithm [1] has been modified and converted into a hardware design built into a CMOS imager with a recognition rate of 96%. The algorithm consists of three steps: smoothing, feature extraction, and lane boundary detection. They are described in detail in this section.

### 2.1. Gaussian Smoothing

In the first step, we remove the noise by applying a Gaussian filter. The one dimensional (1-D) Gaussian filter we use in this paper can be expressed as follows:

(1) $$G(x)=\frac{1}{\sqrt{2\pi }\sigma }e^{\left(\frac{-x^{2}}{2\cdot \sigma^{2}}\right)}$$

where *x* is a variable and σ is the variance of the Gaussian distribution. To perform image convolution with the use of the Gaussian filter, we should digitize the distribution to a discrete form. Empirically, we choose σ=1 to digitize the distribution and obtain a modified 1-D mask as follows.

(2) $$\frac{1}{16}\begin{array}{c} \begin{array}{lllll} 1 & 4 & 6 & 4 & 1 \end{array} \end{array}$$

**Figure 2.**
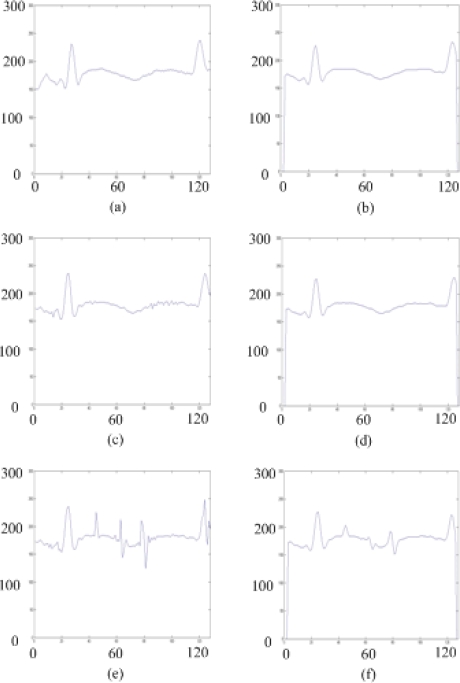
(a), (c), and (e) are the intensity profiles of the original image with pepper noise; and (b), (d), and (f) are the profiles without noise.

Figure 2 (a)(c)(e) show the intensity profiles of a specific row in a original image with pepper noise of 0%, 0.5%, and 1%, respectively. The profiles resulting from the smoothed images by the modified 1-D Gaussian filter are shown in Figure 2 (b)(d)(f).

### 2.2. Feature Extraction

In general, lane markers in the image have two properties: brightness and slenderness. Brightness means that the intensity of the lane mark is higher than that of the road surface under any weather conditions. Slenderness guarantees that the appearance of the lane marker at each row in the image is narrow. According to the intensity peak points obtained due to the properties of brightness and slenderness, the position of the lane marker in the image can be found by analyzing and accumulating the intensity peak points row by row.

**Figure 3.**
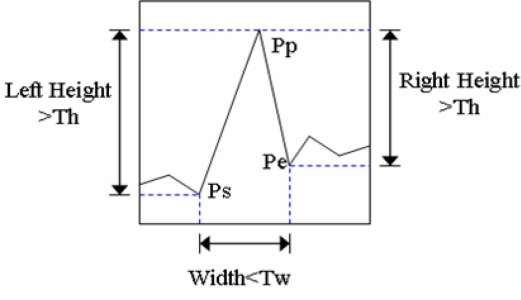
A schematic form describes a hill associating with three variables, *Ps, Pe*, and *Pp*.

The adopted peak-finding algorithm is modified to fit with the hardware requirements in analog design and applied to identify all hill candidates row by row. A hill can be formally defined as a range over which the values increase first and then decrease next without any internal ripples in the profile. Every hill candidate is associated with three variables as shown in Figure 3. *Ps* is the start position of first climbing up point; *Pe* is the end position of last climbing down point; and *Pp* is the peak position of first climbing down point. Then, we define *Left_Height = Pp(y)-Ps(y)*, *Right_Height = Pp(y)-Pe(y)*, and *Width = Pe(x)-Ps(x)*.

The hill candidate that satisfies the following three conditions is considered a hill and the midpoint of the hill is taken as the peak:

$$\begin{array}{l} \mathrm{Left}\_\mathrm{Height}>T_{h}\;\text{and}\\ \mathrm{Right}\_\mathrm{Height}>T_{h}\;\text{and}\\ \mathrm{Width}<T_{w}, \end{array}$$

where *T_h_* and *T_w_* are two thresholds of the hill height and the hill width, respectively. In [1], the values of *T_h_* and *T_w_* are determined empirically and considered fixed. As a result, the peak-finding algorithm is sensitive to image quality. Therefore, we design a circuit which is described later in Section *3* to decide the thresholds automatically. All peaks in an image are the feature points for further lane boundary detection. The image exhibiting all extracted peaks is called the peak-point image.

### 2.3. Lane Grouping

We gather the peaks adjacent to each other to form a line segment. Intuitively, a line segment physically corresponds to one lane marker. In this step, some false peaks resulting from shadows or overpasses will be filtered out. To achieve this, a hardware module referring to as lane-point allocation module is designed and implemented to perform the noise filtering operation. The detailed functionality of lane-point allocation module is described in Section 3.4. Finally, we find line segments to perform the line-segment combination algorithm, which combines the adjacent line segments one by one to obtain the lane boundaries.

## 3. Architecture and Circuit Descriptions

**Figure 4.**
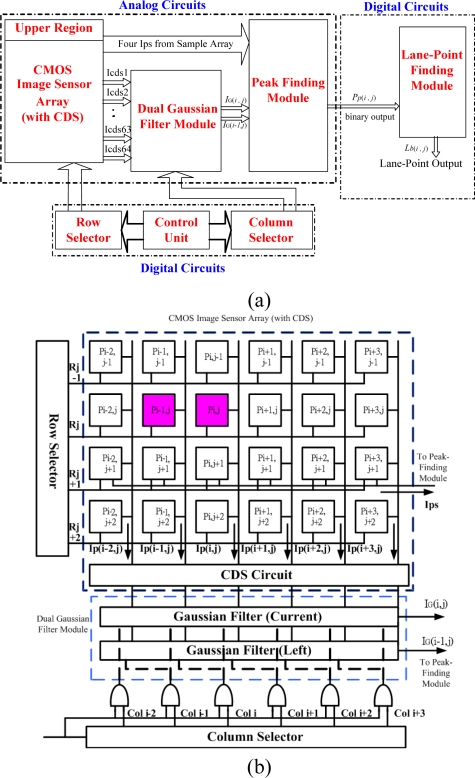
The proposed architectural circuit (a) block diagram (b) signal flow diagram of the proposed mixed signal CMOS imager integrated with lane detection algorithm.

The developed chip as presented Figure 4(a) can be divided into two parts - analog circuits and digital circuits. The analog circuits include a 2-D pixel cell array, a Correlated Double Sampling (CDS) module, a Gaussian filter module, and a Peak-Finding module, whereas the digital circuits comprise a Line Point Allocation module, a column selector, and a row selector. Figure 4(b) gives a detailed explanation of the block diagram in Figure 4(a). The signal flow between the blocks and the whole circuit architecture are presented in these two subfigures.

The peak-finding algorithm extracts the 1-D image intensity profile and locates the peak points at the maximal value with no internal ripple. If the original algorithm is directly used to implement the circuits, an Analog to Digital (A/D) converter is required to convert the analogue current into discrete gray-level values. An additional complicated digital Application Specific Integrated Circuit (ASIC) design should be integrated to process the binary data for gray-level values, increasing the complexity of the system and the consumption of hardware resources to a great extent.

### 3.1. Pixel Cells and Sensor Array

The proposed sensor array prototype is composed of 66 × 66 pixel cells, which comprise 64 × 64 effective pixels. The road and the lane lines are typically located in the bottom part of the processed image. This observation indicates that the top one fourth area of the analyzed image contains sky and cloud. To save hardware resource, the array circuit in this area has no function for the peak-finding operation. The sensor array was divided into two regions: upper and lower. The upper region of 16 × 64 pixels is neglected during the process of lane pixels finding by automatically setting the intensity to “0”. Zero intensity means bypassing the pixel readout operation. The total number of readout operations is reduced to increase the computing efficiency, where the readout speed depends on the simulated clock rate. The lower region is horizontally partitioned into three sub-regions. Each sub-region consists of 16 rows. The 12^th^ row of every sub-region is replaced by a 1-D sampling array. The upper region also has one sampling array in the same manner. Consequently, the sensor array has four 1-D sampling arrays. One is embedded in the upper region and the other three in the lower region. The currents of each sampling array are accumulated in the peak-finding module from which the mean current was collected.

**Figure 5.**
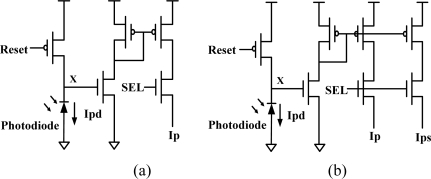
Two types of pixel cell (a) normal cell (b) sampling cell.

**Figure 6.**
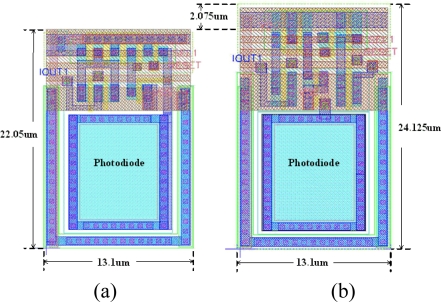
Layout of pixel cell (a) normal cell (b) sampling cell.

The developed sensor array contains two types of pixel cells, as shown in Figure 5. The pixel cell that belongs to the sampling array is called the sampling pixel cell, which consists of seven transistors. The other cell is referred to the normal cell, which comprises five transistors and is smaller than the sampling pixel cell. Hence, there are totally 62 rows of normal cells and 4 rows of sampling cells in the proposed 66 × 66 sensor array. Figure 6 shows the layout diagram of both of the pixel cells. To avoid interference through P-type layer between readout transistors and photodiode, we connect the guard ring of P-type layer to the ground. The width of the sampling cell layout was the same size as the normal cell layout in order to combine both types of the cell layout into the whole 2D sensor array row by row.

**Figure 7.**
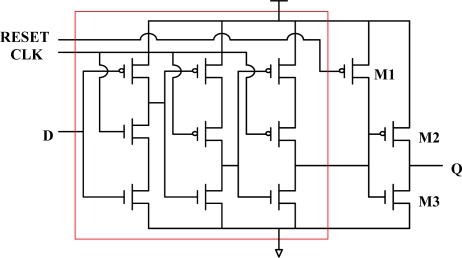
Modified DFF with three extra transistors, M1, M2 and M3.

In this work, the Row/Column selector that addresses and guides the pixel values from the sensor array to the Gaussian filter module includes 64 D-type Flip Flops (DFFs). The True-Signal-Phase-Clocked (TSPC) DFF [11–12, 15] was modified by adding three transistors to obtain one reset function, as shown in Figure 7. The adopted TSPC DFF can be triggered by double edges rather than triggered by positive or negative edge. Also, the total number of transistors in a TSPC DFF is less than that in the popular DFFs. This benefits a high capacity design of a larger sensor array. Consequently, with most of the original features of the adopted TSPC DFF, our proposed design satisfies the requirements of large capacity, high speed, and high extensibility to favour the design of a larger sensor array in the future. The extension of sensor array may be from 64 × 64 to 640 × 480 or to 320 × 240. The common used resolution and the software processing speed for lane detection based on embedded system platform are 320 × 240 and 10 fps, respectively.

### 3.2. Dual 1-D Gaussian Filters

**Figure 8.**
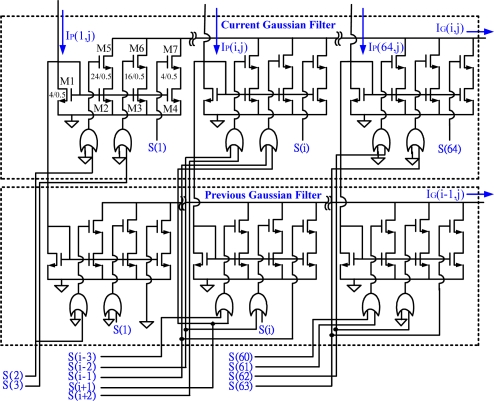
Proposed analogue circuit of the dual 1-D Gaussian filters. The channel width ratio of transistors M4, M3, and M2 (or M7, M6, and M5) is 1:4:6, from right to left.

The developed analogue circuit for a 1-D Gaussian filter includes 64 Gaussian mask cells and a current divider. The 1-D Gaussian filter module includes a couple of 1-D Gaussian filters as shown in Figure 8. The filter smoothes the selected pixel by considering its right and left neighbors and then eliminates noisy points from the original image. Each Gaussian mask cell consists of seven transistors and two OR gates. In the proposed design, the channel width ratio of transistors M4, M3, and M2 (or M7, M6, and M5) is 1:4:6, from right to left, as shown in the upper-left Gaussian mask cell in Figure 8. The relative W/L size of the M4, M3, and M2 transistors are 4/0.5, 16/0.5, and 24/0.5, respectively. The control unit which coordinates the counting and clocking operations of the row and column selectors is shown in Figures 4(a) and (b). It also monitors the timing sequences between the dual filtering units. The operation of the left and the current Gaussian filter successfully provides a modified 1-D mask smoothing function of 1:4:6:4:1 as described in Eqn. (2). The smoothed results, *I_G_(i,j)* and *I_G_(i−1,j)*, output from the dual 1-D Gaussian filters, are integrated into the peak-finding module as shown in Figure 4.

### 3.3. Peak-Finding Module with Auto-Regulated Threshold

**Figure 9.**
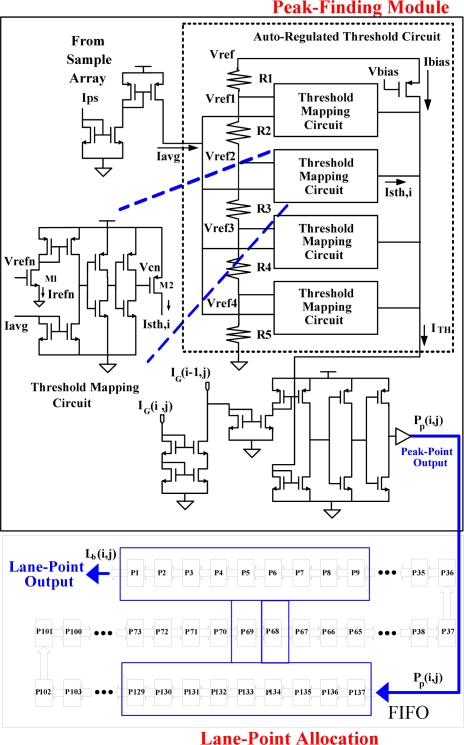
The Proposed Peak-Finding and Lane-Point Allocation Modules.

Extension of the analogue design of current-mode comparator [13–14, 16] enables the peak-finding module, shown in Figure 9, to accumulate and average currents from the aforementioned sampling arrays. Additionally, this module can simultaneously regulate the threshold value and extract peak value. The averaged current from the sampling array, *I_avg_*, was generated according to Eqn. (3):

(3) $$I_{\mathrm{avg}}=\frac{1}{n}\sum_{i=0,j=1}^{n-1,N}\mathrm{Ip}(S+(N/n)*i,j),$$

where *n* is the number of sampling arrays and also indicates the number of the sub-regions in the sensor array. Moreover, N is the total number of rows in the designed imager. S represents the 12^th^ row in each sub-region. Finally, *I_p_* presents the output current from the pixel cells of one sampling array. In this work, *n* and N are set to 4 and 64, respectively.

The auto-regulated threshold circuit compares *I_avg_* with four preconfigured currents to determine the threshold current accordingly. In Figure 9, the above-mentioned functional sub-module includes a voltage divider and four-threshold current mapping circuits. The reference voltage, *V_ref_*, was partitioned by Ri, i=1, 2, 3, 4, and 5, to control four Threshold Mapping Circuits (TMCs). Each TMC consists of a current comparator [13–14,16] and two additional transistors. The transistor *M1* sends various *I_refn_* to the current comparator, as specified by Eqn. (4), depending on *V_refn_* applied to the threshold mapping circuit. If *I_refn_* exceeds *I_avg_*, then *V_Cn_* will be high and *M2* is turned on. This happens whenever the sub-threshold current, *I_sth_*, increases. If *I_renf_* is less than *_Iavg_*, then the *V_Cn_* will be low and *M2* is turned off. Similarly, it happens whenever *I_sth_* approaches to a relatively low value:

$$V_{\mathrm{Cn}}=\{\begin{array}{c} 3.3V,\;\mathrm{if}\;I_{\mathrm{refn}}\geq I_{\mathrm{avg}}\\ 0V,\;\mathrm{otherwise} \end{array}$$

$$V_{\mathrm{refn}}=\left(\frac{\sum_{m=n+1}^{5}\mathrm{Rm}}{\sum_{m=1}^{5}\mathrm{Rm}}\right)V_{\mathrm{ref}}$$

(4) $$I_{\mathrm{refn}}=\frac{1}{2}\frac{W_{M1}}{L_{M1}}u_{n}C_{0}{\left((V_{\mathrm{refn}}-V_{S})-V_{T}\right)}^{2},$$

where n can be 1, 2, 3 or 4, and *V_T_*, W_M1_, L_M1_, *u_n_*, and *c*_0_ are the threshold voltage, channel width, channel length, carrier mobility, and gate capacity of M1, respectively.

The threshold current *I_TH_* is determined by the summation of four TMCs inside the auto-regulated threshold circuit indicated in Figure 9. Equation (5) presents the calculation of the four sub-threshold currents, *I_sth,i_*, where *i* = 0, 1, 2, and 3. In this situation, each M2 of the TMC should be operated in saturation:

(5) $$\begin{array}{l} I_{\mathrm{TH}}=I_{\mathrm{bias}}+\sum_{i=0}^{M-1}I_{\mathrm{sth},i},\;\text{where M is the total number of TMCs and}\\ I_{\mathrm{sth}},i=\frac{1}{2}\frac{W_{M2}}{L_{M2}}u_{n}C_{0}{\left((V_{\mathrm{DD}}-V_{\mathrm{Cn}})-V_{T}\right)}^{2} \end{array}$$

The threshold for extracting the features of lane markers is automatically determined by the hardware module of Auto-Regulated Threshold Circuit (ARTC), as shown in Figure 9 as well. The proposed sampling pixel cell shown in Figure 5(b) together with the normal cell shown in Figure 5(a) are combined into one couple of circuit cells for one image pixel design. This is a new integrated scheme, which has never appeared in the literature as far as we know. It provides I_ps_ to ARTC. The I_ps_ reflects the background picture information so that the adaptive threshold can be tuned automatically. Another current comparator decides whether the current value of the pixel exceeds the threshold value, revealing the existence of a peak point. If the current pixel is a peak point, then the output value will be 1. Otherwise, it should be 0, as presented in Eqn. (6):

(6) $$P_{p}(i,j)=\{\begin{array}{ll} 1, & \mathrm{if}\;I_{G}(i,j)\geq I_{\mathrm{TH}}+I_{G}(i-1,j)\\ 0, & \mathrm{otherwise} \end{array}$$

where *I_G_*(*i,j*) and *I_G_*(*i*−*1,j*) are the current and the previous values output by the dual 1-D Gaussian filters, respectively.

### 3.4. Lane-Point Allocation

In the final processing stage, two digital functions are combined in a single equation, Eqn. (7) to find the lane markers. In this work, we consider the lane marker as the line segment forming by adjacent peak points in different rows. The Lane-Point Allocation module implementing the Eqn. (7) filters out some spurious peak points and selects the correct ones to form the lane markers:

(7) $$L_{b}\left(i,j\right)=\bar{P_{p}\left(i-1,j\right)}•P_{p}\left(i,j\right)•[\sum_{n=-3}^{3}P_{p}\left(i-n,j-1\right)+\sum_{m=-3}^{3}P_{p}(i-m,j+1)]$$

The purpose of the first term in the right-hand side of Eqn. (7) is to filter out the peak point at (*i*, *j*) whose left adjacent point is labeled as a peak point, that is, *P_p_*(*i* − 1, *j*) =1. This preserves the resulting lane marker is one-pixel-width. The last term of Eqn. (7) verifies whether the peak point is at the lane marker or just spuriously isolated one resulting from noise.

**Figure 10.**
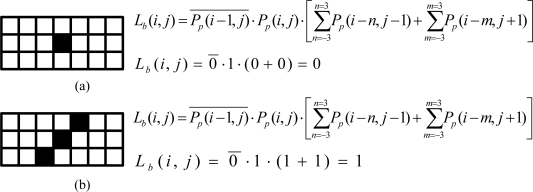
Two examples for evaluating *L_b_*(*i*, *j*) in Lane-Point Allocation Modules (a) example for *L_b_*=0 (b) example for *L_b_*=1.

For covering all possible lane markers with different slopes, a 3 × 7 mask centered at the peak point (*i*, *j*) is used to check the existence of other peak points within this mask either at the row (*j* − 1) or (*j* + 1). If no adjacent peak points exist, the last term of Eqn. (7) will be zero and *L_b_*(*i*, *j*) = 0 which indicates this pixel does not belong to the lane marker. The module with one First-In-First-Out (FIFO) to perform such functionality is shown in the lower part of Figure 9. In order to detail the evaluation of *L_b_*(*i*, *j*), two examples that illustrate different results are shown in Figures 10(a) and 10(b), respectively. The black squares in both examples are the obtained peak points.

## 4. Experimental Results

**Figure 11.**
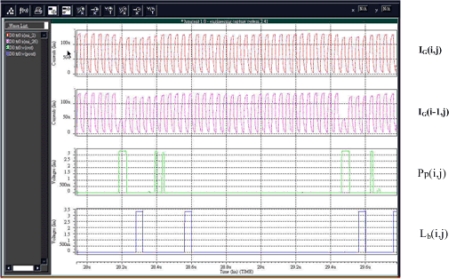
Simulation results for I_G_(i,j), I_G_(i−1,j), P_p_(i,j), and L_b_(i,j).

Images from the real world were captured as testing images to perform the simulation thus confirm the functionality of the proposed chip. Figure 10 displays the simulation results of the key signals in Section III in four windows. The pictures at the first two rows of Figure 11 are the simulation results for current variation of the sample I_G_(i,j) and the previous sample I_G_(i−1,j) after dual 1-D Gaussian filters, respectively. The picture at the third row of Figure 11 presents the binary output results concerning the peak points, Pp(i,j), generated by the peak-finding module and the binary signals that represent the detected lane points produced from the Lane-Point Allocation module in the final stage are shown in the picture at the last row of Figure 11.

**Figure 12.**
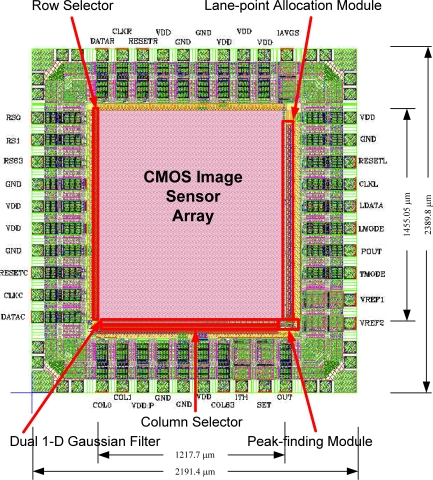
Layout diagram of the proposed CMOS imager.

The developed chip was manufactured in the TSMC 0.35 μm mixed signal process of 2P4M CMOS technology. NMOS capacitors are added to the power line layout regions to reduce power noise. Figure 12 displays the layout of this chip, where the chip size is 2191.4 μm(H) × 2389.8 μm(V) and the image size of the pixel array is 1217.7 μm(H) × 1455.05 μm(V), respectively.

**Figure 13.**
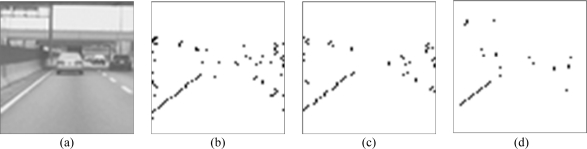
Comparison of experimental results (a) Raw image; (b) is the result of the peak-finding module; (c) is the result of the proposed CMOS imager; (d) is the result of software implementation.

The images in Figure 13 are used to validate the outcome of the proposed CMOS imager. We apply the adopted lane detection algorithm to detect the lane boundaries in C/C++ programming and the resulting image is shown in Figure 13(d). The output of the algorithm contains a few noisy pixels but most pixels on the right lane boundary are missed in the detection processing. Figure 13(b) is the result of the peak-finding module. Obviously, the use of auto-regulated threshold successfully extracts the peak points of the right lane markers. Figure 13(c) is the result of lane boundary map from the lane-point allocation module in the developed CMOS imager, which successfully detects better lane boundaries on both sides only at the expense of introducing a few noisy pixels. The sum of all these building blocks is similar to that of the algorithmic result.

Metal 4 is used as the shield layer to protect the layout areas, except for those regions for photodiodes against etching caused by light effect. Also metal 4 was used as the power line for a smaller current drop, which results from the low resistance effect of the metal 4. Some redundant

The dual pixel cell design with background image information capturing function to automatically control the threshold currents is an innovative idea of this paper. The threshold values in [1] were given manually by expert. Because the manually given expert values are usually better than the automatically generated threshold currents, the detection rate of the proposed imager (less than 96%) is then somewhat worse than that of the software in [1]. On the other hand, in Figure 12(c), more feature points of the right lane boundaries which compare to those in the C/C++ version (see Figure 12(b)) are detected, but with noisier feature points. This means that the developed hardware can occasionally obtain better results in some scenes.

Table 1 gives the design parameters of the developed CMOS imager. The dimensions of the chip are 2191.4 μm × 2389.8 μm, and the package is in a 40-pin DIP. The pixel cell size is 18.45 μm × 21.8 μm and the size of the core of the photodiode is 12.45 μm × 9.6 μm. Also, the resulting fill factor is 29.7%.

## 5. Conclusions

A current-mode mixed signal design of the CMOS image sensor with an integrated peak-finding-based lane detection algorithm is developed herein. This investigation includes a 2-D normal sensor array embedded with four additional modularized circuits for use in smart vehicles. There are four 1-D sample arrays for accumulating the sample currents to adapt the background picture information accordingly; dual 1-D Gaussian filters coupled as an analogue image-smoothing module; an analogue peak-finding function associated with a novel auto-regulated threshold operation, and a FIFO based digital scheme for lane-point allocation.

Most CMOS imager designs have been proposed with a focus on high resolution, high dynamic range, and noise suppression, rather than embedded integration for particular applications. In this investigation, we have developed a low-cost and SoC-based compact chip prototype of CMOS imager that can capture images of roads form the real world and simultaneously identify the lane markers under various weather conditions in real time. To our knowledge, this idea and the proposed prototype chip have never appeared in the previous literature.

## Figures and Tables

**Table 1.** Specification of the proposed CMOS image sensor.

**Item**	**Values**
Effective pixel Count	64 (H) × 64 (V)
Pixel Size	13.1 μm (H) × 22.05 μm (V)
Aperture Size	9.6 μm (H) × 12.45 μm (V)
Fill Factor	29.7 %
Image Size	1217.7 μm (H) × 1455.05 μm (V)
Chip Size	2191.4 μm (H) × 2389.8 μm (V)
Operation Clock	25 MHz
Operation Voltage	3.3 V
Power consumption	159.4 mW
